# Supplementary material for: From sequence to enzyme mechanism using multi-label machine learning
Source: BMC Bioinformatics. 2014 May 19;15:150. doi: 10.1186/1471-2105-15-150 (PMC4229970; doi:10.1186/1471-2105-15-150)
Supplement: Additional file 2 — Java code of ml2db. Additional file ml2db_code.tar.gz contains the Java source code to run the multi-label machine learning experiments and save the results to database. The code’s Javadoc is included. [file 1471-2105-15-150-S2.zip › additional file 2/ml2db/utils/doc/uk/ac/ed/inf/utils/NumberUtils.html]

NumberUtils


---


|  |  |  |
| --- | --- | --- |
| **Overview** \| **Package** \| **Class** \| **Use** \| **Tree** \| **Deprecated** \| **Index** \| **Help** | |  |
| **PREV CLASS**   **NEXT CLASS** | **FRAMES**    **NO FRAMES**     **All Classes** |  |
| SUMMARY: NESTED \| FIELD \| CONSTR \| METHOD | DETAIL: FIELD \| CONSTR \| METHOD |  |


---


## uk.ac.ed.inf.utils Class NumberUtils

```
java.lang.Object
  uk.ac.ed.inf.utils.NumberUtils
```

---

``` public class NumberUtils extends java.lang.Object ```

Class

**Version:**
:   22 Jan 2009

**Author:**
:   Luna De Ferrari luna.deferrari-at-ed.ac.uk

---

**Constructor Summary**

`NumberUtils()`


| **Method Summary** | |
| --- | --- |
| `static java.lang.String` | `doubleTo2DecimalsString(java.lang.Double aDouble)`             Get a percentage representation of a double |
| `static java.lang.String` | `formatPercentOneFraction(double num)`             Takes a double with any number of decimals and returns a String representation of a percentage 0.85842 -> 85.8% |
| `static java.lang.String` | `getValue(java.lang.Double value)`             Gets a string value usable for SQL DOUBLE column. |
| `static boolean` | `isMultipleOf(int multiple, int divisor)`             checks if a number is a multiple of another number. |

**Methods inherited from class java.lang.Object**

`equals, getClass, hashCode, notify, notifyAll, toString, wait, wait, wait`

**Constructor Detail**

### NumberUtils

```
public NumberUtils()
```


**Method Detail**

### doubleTo2DecimalsString

```
public static java.lang.String doubleTo2DecimalsString(java.lang.Double aDouble)
```

:   Get a percentage representation of a double

    :   **Parameters:**: `fraction` - e.g. 0.75 **Returns:**: a percentage, e.g. 75% public static String getPercentage(Double fraction){ Double percent = new Double(fraction); NumberFormat percentFormatter = NumberFormat.getPercentInstance(Locale.getDefault()); return percentFormatter.format(percent); }

---


### formatPercentOneFraction

```
public static java.lang.String formatPercentOneFraction(double num)
```

:   Takes a double with any number of decimals and returns a String
    representation of a percentage 0.85842 -> 85.8%

---


### getValue

```
public static java.lang.String getValue(java.lang.Double value)
```

:   Gets a string value usable for SQL DOUBLE column. The string must be
    either double precision numeric or null: NaN or +/-inf are not acceptable
    values.

    :   **Parameters:**: `the` - value **Returns:**: the double precision value string or null if = NaN or = +/-inf

---


### isMultipleOf

```
public static boolean isMultipleOf(int multiple,
                                   int divisor)
```

:   checks if a number is a multiple of another number. 0 always returns true.

    :   **Parameters:**: `step` -: `i` - **Returns:**


---


|  |  |  |
| --- | --- | --- |
| **Overview** \| **Package** \| **Class** \| **Use** \| **Tree** \| **Deprecated** \| **Index** \| **Help** | |  |
| **PREV CLASS**   **NEXT CLASS** | **FRAMES**    **NO FRAMES**     **All Classes** |  |
| SUMMARY: NESTED \| FIELD \| CONSTR \| METHOD | DETAIL: FIELD \| CONSTR \| METHOD |  |


---
